# Repurposing Antiretroviral Drugs for Urological Cancers: Differential Effects of Protease Inhibitors and NNRTIs on Prostate and Bladder Cancer Cells

**DOI:** 10.3390/cells15121045

**Published:** 2026-06-07

**Authors:** Mariana Pereira, Nuno Vale

**Affiliations:** 1 PerMed Research Group, RISE-Health, Faculty of Medicine, University of Porto, 4200-319 Porto, Portugal; mariana.m.pereira2097@gmail.com; 2 ICBAS—School of Medicine and Biomedical Sciences, University of Porto, Rua de Jorge Viterbo Ferreira, 228, 4050-313 Porto, Portugal; 3 RISE-Health, Department of Community Medicine, Health Information and Decision (MEDCIDS), Faculty of Medicine, University of Porto, Rua Doutor Plácido da Costa, 4200-450 Porto, Portugal; 4 Laboratory of Personalized Medicine, Department of Community Medicine, Health Information and Decision (MEDCIDS), Faculty of Medicine, University of Porto, Rua Doutor Plácido da Costa, 4200-450 Porto, Portugal

**Keywords:** drug repurposing, urological cancer, antiretrovirals, cancer therapy

## Abstract

**Highlights:**

**What are the main findings?**

- Protease inhibitors (ritonavir and saquinavir) showed stronger antiproliferative and anti-clonogenic effects than the NNRTI rilpivirine in prostate (PC-3) and bladder (UM-UC-5) cancer cells.
- Antiretroviral drugs had limited effects on cell migration but modulated intracellular ROS levels in a cell-dependent manner, with low toxicity in healthy cells.

**What are the implications of the main findings?**

- Antiretroviral drugs may preferentially target cancer cell-related proliferation and long-term survival rather than migration capacity.
- Their effects are concentration- and cell-line-dependent.

**Abstract:**

Drug repurposing presents as a promising strategy in oncology, particularly for urological prostate and bladder cancers, where resistance to current therapy remains a challenge. This study evaluated the anticancer potential of three antiretroviral drugs, namely ritonavir (RIT), saquinavir (SAQ), and rilpivirine (RPV), in PC-3 and UM-UC-5 cancer cell lines, using MTT, clonogenic, wound healing, toxicity assessment with fibroblast cells, and DCFDA assays; this last method included efavirenz (EFV) and etravirine (ETV) for intracellular reactive oxygen species (ROS) production. RIT and SAQ showed stronger antiproliferative effects than RPV, with lower concentration- and cell-line-dependent activity, while clonogenic assays confirmed a reduction in long-term proliferation, particularly for RIT in both cell lines and SAQ for UM-UC-5. In contrast, effects on cell migration were limited for all drugs. ROS production was cell-dependent, with EFV increasing ROS in PC-3 and SAQ and RIT in UM-UC-5 cells. Generally, all drugs showed minimal toxicity in non-malignant cells, with SAQ exhibiting some toxicity but only for concentrations higher than those required for anticancer activity. Overall, these findings suggest that antiretroviral, especially protease inhibitors, may cause anticancer effects, although these are concentration- and context-dependent, and further investigation is needed to understand the mechanisms involved.

## 1. Introduction

Although considerable progress has been made in cancer diagnosis and treatment, this disease remains a major worldwide health burden, with high incidence and mortality [1]. Among urological malignancies, prostate and bladder cancer still have a high prevalence and mortality rates, particularly due to disease progression to metastasis and development of resistance to therapy [2]. This highlights the need for alternative therapeutic approaches capable of complementing or improving current treatment regimens.

Drug repurposing has emerged as a novel strategy in oncology, since it allows existing approved drugs to be redirected for new therapeutic indications. This not only reduces both costs and time spent, while benefiting from already established pharmacokinetic, pharmacodynamic, and safety profiles [3]. In the context of cancer, several non-oncological drugs have shown anticancer properties, including the ability to decrease cell proliferation [4], induce apoptosis [5], and promote cell-cycle arrest [6], among other effects.

Antiretroviral drugs, originally developed for the treatment of human immunodeficiency virus (HIV) infection, have been shown to possess anticancer effects [7,8]. Protease inhibitors (PIs), including ritonavir (RIT) [9] and saquinavir (SAQ) [10], as well as non-nucleoside reverse transcriptase inhibitors (NNRTIs) like rilpivirine (RPV) [11], etravirine (ETV) [12], and efavirenz (EFV) [13], have been reported to modulate several tumor-associated cellular processes [14,15]. These included the induction of oxidative stress [16], apoptosis [17,18], cell cycle arrest [19], and impairment of cell migration [20].

This work aimed to further characterize the effects of RIT, RPV, and SAQ in bladder and prostate cancer cell models in various aspects associated with tumor progression, such as cell migration and clonogenic potential. Additionally, the effects of these drugs, together with EFV and ETV, on intracellular reactive oxygen (ROS) levels were investigated. In parallel, the toxicity of these drugs in non-malignant cells was evaluated to give a preliminary indication of the safety of these drugs. Collectively, these analyses focused on exploring the potential of antiretroviral drugs as candidates for cancer repurposing in prostate and bladder cancer.

The selection of RIT, SAQ, and RPV was carried out based on both pharmacological class and previous anticancer research. SAQ and RPV have been investigated by this research group in prostate [21] and bladder cancer models [22], respectively, where both drugs demonstrated antiproliferative activity. The present study expands on these findings by evaluating these drugs on the complementary urological cancer models, namely SAQ in bladder cancer cells and RPV in prostate cancer cells, while also incorporating new functional assays addressing other hallmarks of cancer. RIT was selected for new testing due to its extensive preclinical evidence supporting anticancer activity and its effects on drug-metabolizing enzymes and transporters inhibition, chemoresistance reversal, and pharmacokinetics boosting of other compounds [23,24,25,26,27]. Although other anti-HIV drug classes, such as nucleoside reverse transcriptase and integrase inhibitors, have also demonstrated anticancer potential [28,29], this study focused on compounds with stronger and more characterized preclinical evidence.

## 2. Materials and Methods

### 2.1. Cell Culture

The effects of RIT, RPV, and SAQ were assessed using three human cell lines: UM-UC-5 (bladder cancer), PC-3 (metastatic prostate cancer), and MRC-5 (fetal lung fibroblast). All cell lines were purchased from the American Type Culture Collection (ATCC, Manassas, VA, USA). PC-3 cells were selected because they are a androgen-independent prostate cancer model representative of advanced disease, widely used [30]. UM-UC-5 cells are a model of primary transitional bladder cancer and were selected due to their known molecular profile and prior use in pharmacologic studies [31,32]. Together, these models were chosen to allow the evaluation of the anticancer activity of HIV drugs in aggressive and relevant urological cancer settings, rather than to represent the biological heterogeneity of prostate and bladder cancer.

Drugs were purchased from Sigma-Aldrich (Merck KGaA, Darmstadt, Germany). Unless stated, all material used in cell culture was purchased from Millipore Sigma (Merck KGaA, Darmstadt, Germany). All procedures were conducted under sterile conditions in a laminar flow chamber, with equipment and materials disinfected with 70% ethanol before use.

Cell culture was carried out in Dulbecco’s modified Eagle’s medium (DMEM), with 1% penicillin–streptomycin solution and 10% fetal bovine serum (FBS), on an incubator at 37 °C and with 5% CO_2_. After reaching confluency, cells were routinely passaged using 0.25% trypsin-EDTA (Gibco, Thermo Fisher Scientific, Inc., Waltham, MA, USA), with culture medium being replenished every 96 h. For experiments, cells were seeded into 96-well plates at well densities of 5000 (UM-UC-5), 3000 (PC-3), and 8000 (MRC-5), and left to adhere overnight.

### 2.2. Drug Treatment

RIT (in PC-3 and UM-UC-5), RPV (in PC-3), and SAQ (in UM-UC-5) were tested in a concentration range that included 0.01, 0.1, 1, 10, 25, 50, and 100 μM over 24, 48, and 72 h. This range was selected to capture a broad response profile and allow the acquisition of reliable IC_50_ values, in accordance with the standard drug repurposing workflow. For toxicity evaluation in healthy cells, concentrations of 10, 25, and 50 μM were applied for 72 h.

Dimethyl sulfoxide (DMSO) was used to solubilize all drugs, which were then diluted into culture medium, giving a final concentration of 0.1% of DMSO. Each condition was tested in three individual experiments, and 0.1% DMSO was used as a vehicle control.

### 2.3. Morphological Analysis

Changes in cell morphology were monitored for each condition using a Leica DMI 6000B microscope coupled with a Leica DFC350 FX camera (Leica Microsystems, Wetzlar, Germany). Images were acquired and analyzed using the Leica LAS X software (v3.7.4; Leica Microsystems, Wetzlar, Germany).

### 2.4. MTT Assay

Cell viability was evaluated through the thiazolyl blue tetrazolium bromide colorimetric assay (MTT). At the end of each treatment, in each well, 100 μL of MTT solution (0.5 mg/mL in PBS, phosphate-buffered saline) was added, and cells were incubated for two hours in the dark (at 37 °C with 5% CO_2_). The formazan purple crystals formed were solubilized with 100 μL of DMSO. Absorbance was measured using a Tecan Infinite M200 microplate reader (Tecan Group Ltd., Männedorf, Switzerland) at 570 nm, and cell viability was calculated as a percentage of the negative control.

### 2.5. Wound Healing (Gap Closure) Assay

Cell migration was evaluated using a wound healing gap closure assay. To generate a uniform and reproducible cell-free gap, silicone inserts (ibidi GmbH, Gräfelfing, Germany) were placed in 12-well plates, avoiding the typical mechanical damage associated with scratch assays. A volume of 110 μL of cells was seeded on all four sides of the inserts, at a concentration of 8 × 10^5^ for UM-UC-5 and 3 × 10^5^ for PC-3 cells, and left to adhere for 24 h. Following insert removal, cells were washed twice with PBS to remove any non-adherent cells, and treatment with RIT, RPV, and SAQ for 48 h was performed. Images of the gaps were acquired at 0, 24, 48, and 72 h, and wound closure was quantified using ImageJ (FIJI, version 1.53, NIH, Bethesda, MD, USA). Results were obtained by comparing the gap area at each time point to the initial gap at hour 0.

### 2.6. Clonogenic Assay

For clonogenic assays, 100 cells per well of UM-UC-5 and PC-3 were seeded in 6-well plates and left to adhere for 24 h. Cells were then treated with RIT, RPV, and SAQ for 48 h. After this time, the medium was replaced with drug-free medium, and cells were allowed to grow for 12 more days, with new medium every 2 days, for a total of 14 days. When this point was reached, colonies were fixed with methanol, stained with a crystal violet solution (0.5% *v*/*v*), and counted manually, including only non-overlapping colonies larger than 0.5 mm. Results were presented as a percentage of control (treated with only 0.1% DMSO).

### 2.7. DCFDA Assay

Intracellular reactive oxygen species (ROS) levels were indirectly evaluated using the DCFDA (2′,7′-dichlorofluorescein diacetate) fluorescent probe in PC-3 (EFV, ETV, SAQ, and RIT) and UM-UC-5 (EFV, SAQ, RIT) cells. Cells were seeded in 96-well black plates and allowed to adhere for 24 h, after which 100 μL of 100 μM DCFDA diluted in PBS was added and incubated in the dark for 30 min at 37 °C. Following this step, the excess probe was removed, the wells were washed, and culture medium with the desired drug concentration was added and incubated for 48 h. After this time, the fluorescence was recorded using a SpectraMax Gemini EM Microplate Reader (Molecular Devices, San Jose, CA, USA), with an excitation wavelength of 485 nm and an emission wavelength of 530 nm.

### 2.8. Statistical Analysis

Data are shown as mean ± SEM, with analysis and graph creation being performed using GraphPad Prism 9 (GraphPad Software Inc., San Diego, CA, USA). Values obtained from experiments were normalized as a percentage of negative controls, and differences between treatment and controls were assessed using one-way ANOVA followed by Dunnett’s multiple comparisons test, with the significance threshold at *p* < 0.05.

## 3. Results

### 3.1. Cell Viability

**Figure 1:**
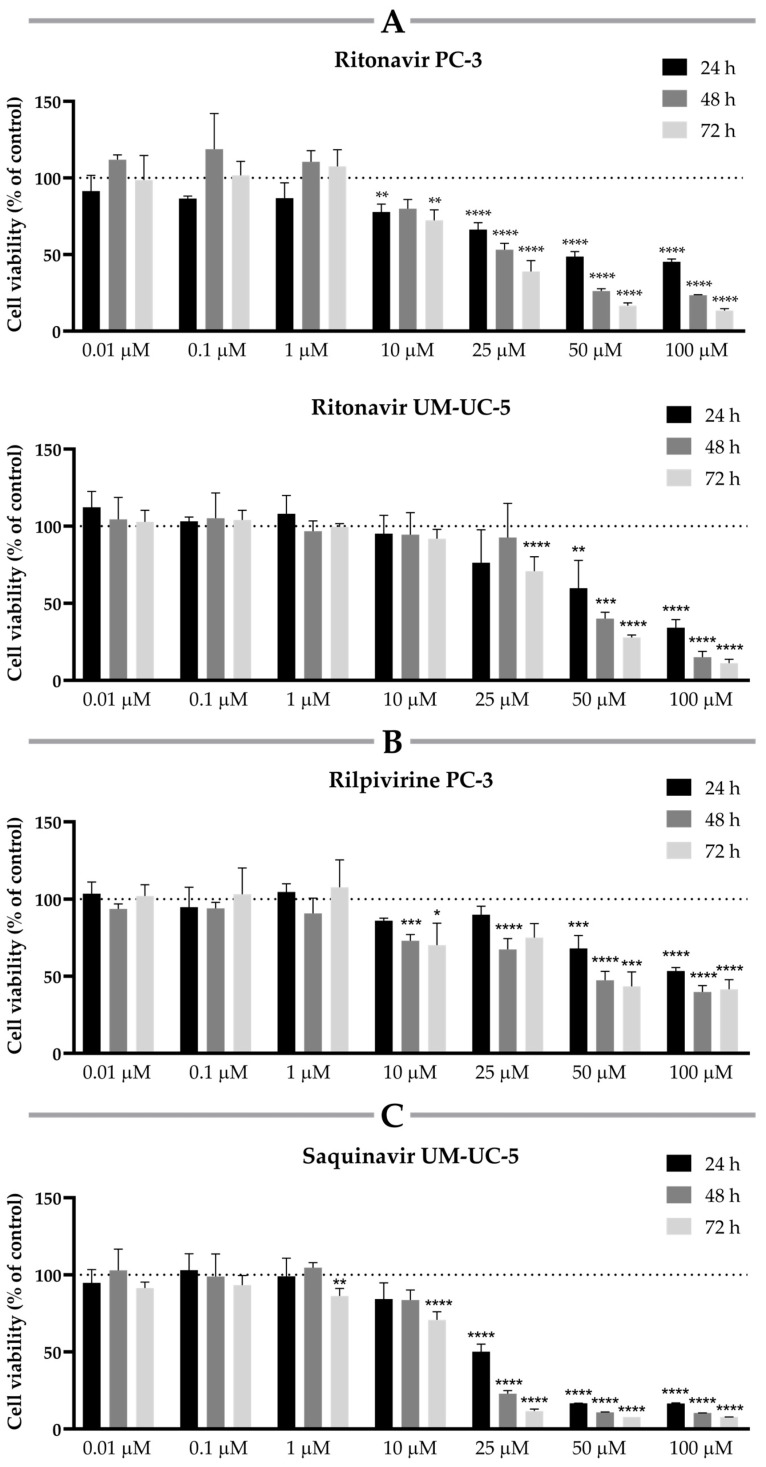
Cytotoxic effect obtained from MTT assays after 24, 48, and 72 h of exposure to the following: (**A**) RIT in PC-3 and UM-UC-5 cells; (**B**) RPV in PC-3 cells; (**C**) SAQ in UM-UC-5 cells. Cells were treated with concentrations ranging from 0.01 to 100 µM, and viability was normalized to the vehicle-treated controls (0.01% DMSO). Data are presented as mean ± SEM of three independent experiments. Statistical significance was determined relative to the negative control (* *p* < 0.05; ** *p* < 0.01; *** *p* < 0.001; **** *p* < 0.0001).

Cell viability was assessed for RIT, RPV, and SAQ. The results are shown in Figure 1, and the IC_50_ values are presented in Table 1.

RIT has been shown to decrease cell viability in a time- and concentration-dependent manner in both PC-3 and UM-UC-5 cell lines. A stronger effect is observed in the prostate cancer cell line, with an IC_50_ below 30 µM at 48 h, which is lower than that of bladder cancer, even at the longest time point (35 µM at 72 h) (Figure 1A).

RPV reduced cell viability in a concentration-dependent manner up to 50 µM, with minimal further decrease beyond that in PC-3 cells. The IC_50_ values at 48 and 72 h are very similar (54.03 and 53.77 µM, respectively), indicating that its effect stabilizes after 48 h (Figure 1B). Compared to its activity in bladder cancer cells, RPV is more effective in prostate cancer, as the lowest IC_50_ for UM-UC-5 cells was around 112 µM [22], observed only at 72 h, which is similar to the IC_50_ at 24 h in PC-3 cells.

SAQ exposure led to a decrease in relative UM-UC-5 cell viability in a concentration- and time-dependent manner. The IC_50_ is already low for 24 h (~25 µM), reducing the longer the exposure time (Figure 1C). These results are slightly better than those for the PC-3 cells obtained in previous work, where IC_50_ values ranged from ~32 to ~21 µM [21], although the difference is not as marked as for RPV.

### 3.2. Drug Safety Assay

**Figure 2:**
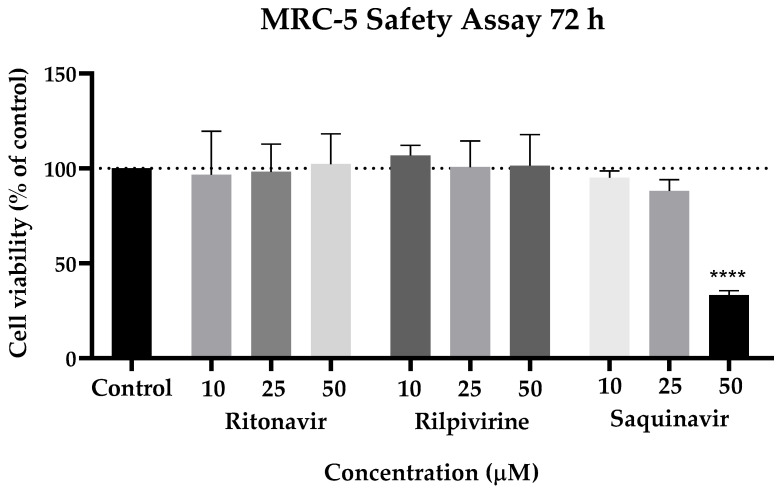
Cell viability results of RIT, RPV, and SAQ in MRC-5 cells obtained from MTT assays for 72 h. Cells were treated with concentrations of 10, 25, and 50 µM, and viability was normalized to the vehicle-treated controls (0.01% DMSO). Data are presented as mean ± SEM of three independent experiments. Statistical significance was determined relative to the negative control (**** *p* < 0.0001).

**Figure 3:**
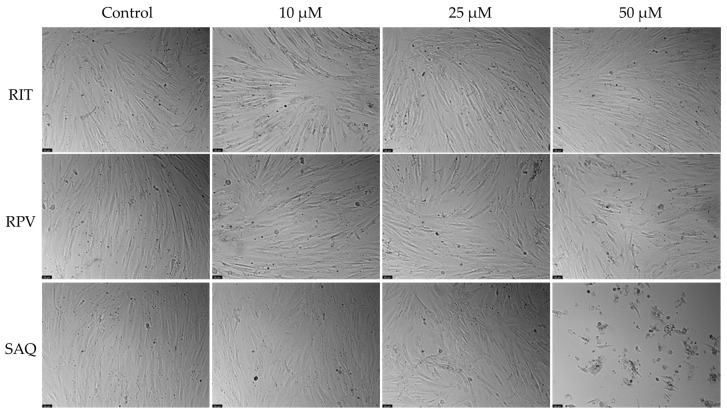
Morphological evaluation of MRC-5 after exposure to 10, 25, and 50 μM of RIT, RPV, and SAQ for 72 h. Control cells were treated with the vehicle (0.01% DMSO). These images are representative of three independent experiments. The scale bar is 50 μm.

Potential toxicity towards non-malignant cells was investigated by exposing the MRC-5 cell line to 10, 25, and 50 µM of each drug for 72 h, followed by viability assessment with the MTT assay. The cell viability graph is shown in Figure 2 and cell morphology in Figure 3. RIT and RPV demonstrated no cytotoxicity for any concentration tested (Figure 2), without any alteration of their natural morphology (Figure 3), so they are considered to be safe.

SAQ started to reduce cell viability at 25 µM, although modestly, with a strong, statistically significant effect at 50 µM, where viability decreased by about 45% (Figure 2). This can also be seen in morphology images, as cells have a much lower abundance and no longer present the elongated form they normally have (Figure 3). However, as the highest IC_50_ obtained for either cell line was ~21 µM, SAQ can be considered safe at the concentrations intended for repurposing.

### 3.3. Wound Healing (Gap Closure) Assay

**Figure 4:**
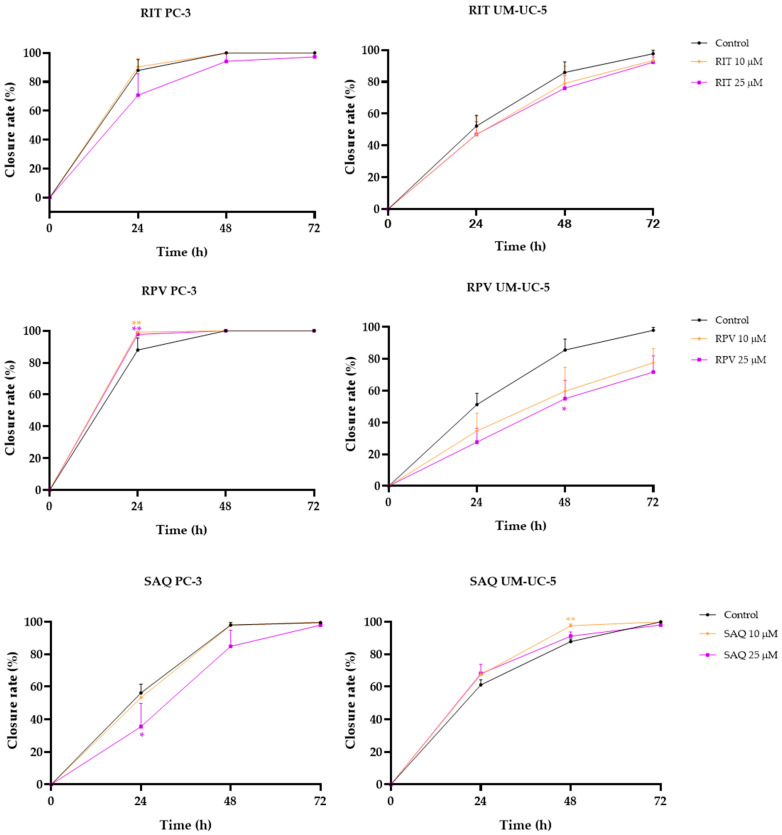
Effects of RIT, RPV, and SAQ on the migration of PC-3 and UM-UC-5 cells were evaluated using the wound healing (gap closure) assay. Cells were treated with 10 and 25 µM of each drug, and images were acquired at 0, 24, 48, and 72 h following insert removal. Gap closure was quantified relative to the initial wound area using ImageJ software and expressed as a percentage of wound closure. Data are presented as mean ± SEM of three independent experiments (* *p* < 0.05; ** *p* < 0.01).

Cell migration was evaluated by measuring gap closure over 72 h after cell treatment with RIT, RPV, and SAQ, and the results are exhibited in Figure 4.

For PC-3 cells, the negative control showed a rapid gap closure, with near-coverage at 48 h. RIT at 10 µM yielded results like the negative control, while 25 µM showed a slight, but not statistically significant, effect only at 24 h (around 20%). SAQ showed a comparable outcome, with 10 µM not altering migration, whereas 25 µM had a significantly slowed closure rate at 24 h, also of around 20%, although this result decreased over time. In contrast, RPV not only failed to impair migration, but in fact accelerated the closure rate at 24 h when compared to negative control, for both concentrations, achieving complete closure at that time point.

In the UM-UC-5 cell line, migration in the negative control was more gradual, with complete closure only at 72 h. RIT had a non-significant reduction in migration that was equal for both concentrations, with results almost matching the negative control. RPV had a more pronounced effect, particularly at 25 µM, with lower results at all time points, but only statistically significant at 48 h, where gap closure was reduced by 20–25% relative to the negative control. Conversely, SAQ had almost no inhibition of migration, with 25 µM having similar results to the negative control, and 10 µM having an acceleration of closure of 10% for 48 h.

Generally, these drugs had weak results in terms of inhibiting cell migration for both cell lines, with the better outcomes being observed for RPV in UM-UC-5 cells.

### 3.4. Colony-Forming Assay

**Figure 5:**
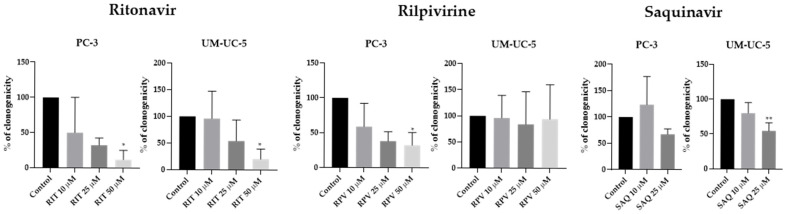
Effects of antiretroviral drugs RIT, RPV, and SAQ in the clonogenic capacity of PC-3 and UM-UC-5 cells after 48 h of exposure to concentrations ranging from 10 to 50 µM. Results are expressed as a percentage of the negative control, and data is given as a mean ± SEM of three independent experiments. Statistical significance was determined relative to the negative control (* *p* < 0.05; ** *p* < 0.01).

The long-term effects of the antiretroviral drugs on colony-forming ability were evaluated in the PC-3 and UM-UC-5 cell lines. Figure 5 shows the graphs obtained for each cell line-drug combination.

In PC-3 cells, RIT had a concentration-dependent decrease in clonogenic capacity, reaching statistical significance for the higher concentration of 50 µM, with reductions of around 70–80%. Similar results were obtained for RPV, but to a lesser extent, of only around 60% reduction. In contrast, SAQ had no significant alteration in the colony-forming capacity of cells.

RIT had a similar effect in UM-UC-5 cells as it did in PC-3 cells, decreasing colony formation, with a significant decrease at 50 µM of around 75%. On the other hand, RPV showed no effects at all, with clonogenic capacity staying like that of the negative control. Also, in contrast to what was observed in the prostate cancer cell line, SAQ significantly impaired colony formation at 25 µM, the highest concentration tested, with a reduction of around 50%, showing a stronger effect of this drug on this cell line.

Overall, the results indicate that RIT is a strong drug in terms of reducing the clonogenic capacity of both cell lines, while RPV and SAQ induced only cell-dependent effects.

### 3.5. Intracellular ROS Production (DCFDA Assay)

**Figure 6:**
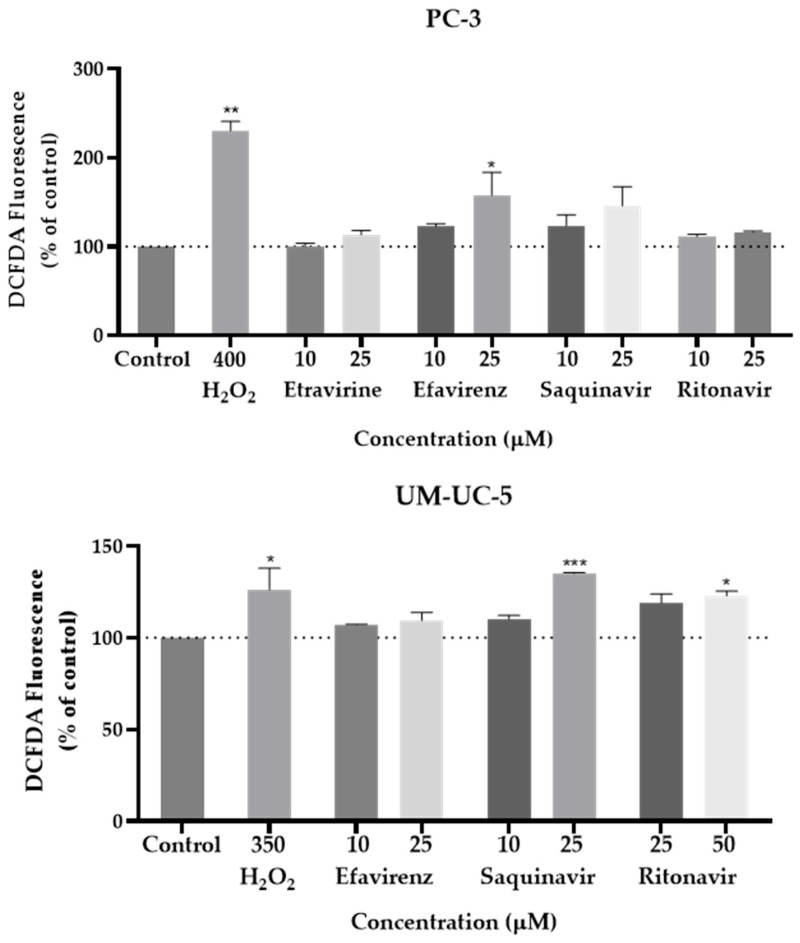
Intracellular ROS production in PC-3 and UM-UC-5 cells was measured using the DCFDA fluorescence assay after exposure to antiretroviral drugs. Cells were treated with the indicated concentrations of EFV, ETV, RIT, or SAQ for 48 h following DCFDA loading. H_2_O_2_ was used as a positive control for ROS induction. Fluorescence values were normalized to vehicle-treated controls and expressed as a percentage of control fluorescence intensity. Data are presented as mean ± SEM of three independent experiments. Statistical significance was determined relative to the negative control (* *p* < 0.05; ** *p* < 0.01; *** *p* < 0.001).

Intracellular ROS levels were assessed using the DCFDA assay in PC-3 and UM-UC-5 following treatment with 10 and 25 µM of each drug, and results are 0 presented in Figure 6. A test with hydrogen peroxide (H_2_O_2_) was carried out to confirm that the assay was working correctly. In both cell lines, H_2_O_2_ exposure showed a statistically significant increase in ROS production.

In the PC-3 cell line, EFV at a concentration of 25 µM induced a significant increase of around 50% in ROS production, with a lower concentration presenting only a modest increase. SAQ had a moderate concentration-dependent, non-significant increase in ROS levels. Neither ETV nor RIT caused alteration in ROS production, with values near baseline (Figure 6, top).

For the UM-UC-5 cell line, and in contrast to the prostate cancer cell line, SAQ at 25 µM induced a significant increase of 30% in ROS production, the strongest effect of all drugs in this cell line. RIT treatment at 50 µM also had a moderate, statistically significant increase of around 20%, while a lower concentration showed a slight increase, but without significance. EFV ROS values stayed close to negative control values (Figure 6, bottom).

These results indicate that some of these drugs modulate intracellular ROS levels in a cell-dependent manner, with EFV having a stronger impact in PC-3 cells, while SAQ had a bigger effect on UM-UC-5 cells, with RIT also showing some increase in this cell line, but only for high concentrations.

## 4. Discussion

In the present study, the characterization of the antiretroviral drugs RIT, SAQ, and RPV in the prostate and bladder cancer models was expanded through the evaluation of additional cancer-related endpoints, including migration, clonogenic potential, and toxicity towards non-malignant cells. In parallel, EFV and ETV were also investigated for their effects on intracellular ROS production. Altogether, the results obtained indicate that these drugs exert cell-dependent effects across multiple functional assays, supporting their potential for repurposing in prostate and bladder cancer.

In terms of antiproliferative effects, the protease inhibitors RIT and SAQ showed higher and more consistent activity than the NNRTI RPV. HIV PIs have been shown to have strong repurposing potential for cancer treatment, generally through the Akt pathway [33], endoplasmic reticulum (ER) stress [34], proteasome inhibition [35], and autophagy [14,36]. All drugs showed limited toxicity in the non-malignant MRC-5 cell line under the concentrations tested. Only SAQ at a higher concentration had negative effects on the viability of this cell line, but those were above the required for antitumoral effect.

RIT showed more potency in PC-3 cells, and the relationship between this PI and prostate cancer has been demonstrated before in several studies. Notably, RIT has been shown to inhibit cell growth and promote apoptosis in androgen-independent prostate cells [37]. In addition, it has also been reported to inhibit CYP3A4 activity and P-glycoprotein-mediated transport in therapy-resistant cells, leading to a reversal of resistance to docetaxel and cabazitaxel, two chemotherapeutic agents used in prostate cancer treatment [26,37]. While higher concentrations of RIT were required to achieve the inhibitory effects in the bladder cancer cell line when compared to the prostate cancer cell line, it still showed efficacy with prolonged exposure. In a recent study, RIT exhibited moderate antiproliferative and apoptotic effects in several bladder cancer cell lines at similarly high concentrations (40 µM), which is consistent with the present findings. The reported accumulation of ubiquitinated proteins suggested that ER stress induction may contribute to the reduction in bladder cancer cell viability following RIT exposure. Despite these results, the study suggested that RIT may be more clinically relevant in combination-based therapeutic strategies for bladder cancer rather than as a monotherapy, since it shows that the anticancer activity is more pronounced when used in combination with the proteasome inhibitor ixazomib [38]. Another mechanism potentially contributing to the observed reduction in cell viability of RIT could be its proven inhibition of the ALKBH2 DNA damage repair enzyme. This enzyme is a human homologue of the bacterial enzyme AlkB2 and is typically overexpressed in tumorigenesis and chemotherapy resistance development [39]. ALKBH2 is overexpressed in bladder cancer [40], and RIT was highlighted in a study as a competitive inhibitory drug of ALKBH2 in vitro, with strong binding affinity, and its use increased the sensitivity of PC-3 ALKBH3 knockdown cells to methylmethane sulfonate, which can be related to that inhibitory effect [27].

SAQ demonstrated a strong antiproliferative effect in UM-UC-5 cells, with IC_50_ values even lower than those of RIT. This research group has previously reported effects on prostate cancer cells, but with slightly higher IC_50_ values [21]. SAQ drug has been previously shown to inhibit 20 s and 26 s proteasome activity in several prostate cancer cell lines at concentrations of 10 µM, while also inducing apoptosis in a concentration-dependent manner and sensitizing prostate cancer cells to ionizing radiation, all associated with inhibition of NF-κB activation [17]. In bladder cancer models, SAQ has also been associated with cell cycle arrest and apoptosis, through the upregulation of survivin-related signaling pathways [41], which may contribute to the cell viability reduction observed in UM-UC-5 cells. SAQ, among other PIs, has also been shown to block Akt activation by phosphorylation in patients receiving the standard dosing of this drug, leading to radiosensitization [42], with evidence also existing for the inhibition of Akt in a cervical cancer progression pathway by this drug [43]. The PI3K/Akt pathway is strongly implicated in multiple cancer hallmarks, including cell survival, metastasis, and angiogenesis [44], and it has also been associated with the reversal of radioresistance in cancer cells [45]. The interference of SAQ with the PI3K/Akt pathway, which has also been reported for RIT [46,47], may represent a mechanism of action of these drugs in bladder and prostate cancer. Notably, the difference between the effective antiproliferative concentrations and the toxic concentration in MRC-5 cells is small (13 vs. 21 µM), which suggests that careful evaluation of therapeutic window and dosing strategies will be required in future translational studies.

In contrast to RIT and SAQ, RPV exhibited less antiproliferative activity in PC-3 cells, with higher IC_50_ values (around 50 µM) and no significant difference beyond 48 h of exposure, indicating a more cytostatic effect. Previously, a study by this group also showed that this drug had little effect in UM-UC-5 cells, with IC_50_ values above 100 µM and stronger effects only for longer exposure times [22]. While both NNRTIs and PIs have shown anticancer potential in preclinical studies, NNRTI-associated effects are generally more context-dependent and are not as validated in cancer models [15]. Potentially, the effect RPV had could be related to Aurora A kinase inhibition, a key regulator of the G2/M transition. This mechanism could contribute to the predominantly cytostatic profile of RPV in PC-3 cells, as they may be accumulating at this stage of the cell cycle [19].

The clonogenic assay showed that the antiproliferative activity observed in the MTT assays was sustained over time, with stronger effects on the same cell lines as those with lower IC_50_ values (RIT and RPV in PC-3, and SAQ in UM-UC-5). SAQ has been reported to inhibit proliferation and clonogenicity in cervical cancer cells (up to 90% colony reduction at 19 µM) [48], while RIT shows anti-clonogenic activity in lung cancer cells (IC_50_ around 30–40 µM, potentially due to cell cycle arrest caused by the reduction in survivin amounts) [49]. RPV inhibits colony formation in pancreatic cell lines (EC_50_s of 16.2 µM) [50]. These results and information in the literature suggest that RIT, RPV, and SAQ have a sustained impact on the proliferative activity of the cancer cell lines, suggesting a reduction in the long-term tumorigenic potential and recurrence [51].

In contrast to the effects observed in the colony-forming assay, the impact of these antiretrovirals on cell migration was weaker. Only RPV had a significant inhibitory effect on UM-UC-5 cells, while RIT and SAQ had limited and transient effects in both models. The results suggest that the tested antiretrovirals tend to interfere with cell growth and long-term survival rather than with the motility of cells. These findings may reflect distinct signaling pathways, as motility is related to cytoskeletal remodeling and cell adhesion [52], while cell viability and colony formation are related to metabolic pathways and growth inhibition [53,54]. Therefore, under the concentrations and exposure times evaluated in this study, the findings indicate that these antiretrovirals may suppress prostate and bladder tumor cell expansion without interfering with their migratory behavior.

Intracellular ROS production was investigated due to previous scholarly evidence showing that oxidative stress has been associated with the anticancer activity of antiretrovirals on other types of cancers [16,55]. The results showed a cell-dependent effect. EFV was included in this study of ROS due to a previous report that this drug can decrease cell viability in both cell lines, with IC_50_ values around 20 µM, decrease colony formation, and inhibit UM-UC-5 cell migration [56]. Additionally, EFV anticancer activity has been associated with ROS formation and oxidative stress before, alone in pancreatic cancer cells [55], and in combination with metformin and fluoxetine in colon cancer cells [57]. In the present work, EFV also increased ROS formation in PC-3 cells, the same cell line with the lowest IC_50_ value, suggesting that oxidative stress modulation may contribute to the anticancer effects of EFV in prostate cancer cells. Increased ROS levels were also detected in SAQ and RIT for UM-UC-5, suggesting that these PIs may cause redox imbalances in bladder cancer cells. These findings are consistent with previous scholarly reports of several HIV PIs, including RIT and SAQ, describing induced proteotoxic stress with consequent apoptosis, and inhibition of pro-survival signaling like Akt-phosphorylation, in acute myeloid cells [58].

Some limitations of this research paper must be mentioned. All experiments were carried out in vitro and using a single cell line for each cancer type, which restricts clinical extrapolation and does not capture the molecular and phenotypic heterogeneity of these urological cancers. Future work should include a broader panel of cancer cell lines, with different invasive and molecular characteristics for bladder cancer, such as RT4 and T24, as well as androgen-responsive prostate cancer models, like LNCaP. Additionally, the use of other models, such as 3D cultures, patient organoids, and in vivo xenografts and animal models, would improve the relevance of these results and the understanding of therapeutic potential and translational applicability.

Another limitation is the relationship between effective in vitro concentrations and clinically achievable plasma concentrations. IC_50_ values and alterations in clonogenicity and ROS production were generally achieved around 10–25 µM, with higher concentrations for RPV. These may exceed plasma concentrations during conventional antiretroviral therapy and could cause off-target effects. These results should, therefore, be interpreted as exploratory preclinical evidence rather than direct support for the use of these drugs as a monotherapy. However, plasma concentrations may not fully predict pharmacological relevance, as intracellular accumulation and tissue distribution may differ from circulating concentration [59,60]. Additionally, as stated above, HIV PIs like SAQ and RIT have shown greater translational potential in combination therapies, where they can increase the anticancer effects of other drugs and lead to radio and chemosensitization [16,26,38,48,58]. Investigation of these antiretrovirals in prostate and bladder cancer would benefit from prioritizing their use in combination and attaining effective concentrations in plasma.

The present study shows an association between drug exposure, ROS modulation, and reduced cancer cell viability and clonogenic potential, but it does not establish a direct causal relationship between these effects. Future work should focus on performing antioxidant rescue experiments to determine whether the oxidative stress caused by SAQ, RIT, and EFV directly contributes to their anticancer activity [61]. Investigation of mitochondrial function and metabolic alterations associated with ROS modulation by these drugs may also provide valuable insight. Additional mechanistic studies should be performed, including apoptosis assays (annexin V/PI staining), cell cycle analysis, and transcriptomic and proteomic characterization of molecules and pathways potentially involved in the observed anticancer effects. These assays could help characterize the mechanisms of action behind the reductions in viability observed in the MTT assay and other anticancer effects.

## 5. Conclusions

In conclusion, the present study highlights the potential of antiretroviral drugs, particularly HIV PIs, to impair prostate and bladder cancer cell viability and long-term proliferative capacity. RIT and SAQ exhibited stronger and more consistent effects than RPV, although responses varied between cell lines. The comparatively limited effects observed in migration assays suggest that these drugs may primarily affect tumor growth and survival rather than cell motility. While results point to the possible contribution of oxidative stress modulation to the observed anticancer effects, further mechanistic research is needed. Overall, these findings support the continued exploration of antiretroviral drugs as potential candidates for prostate and bladder cancer treatment, particularly through more complex models and in mechanistic studies, as well as via combination approaches, to better define their therapeutic relevance.

## Figures and Tables

**Table 1:** IC_50_ values for various time points obtained from MTT assays of antiretroviral drugs in PC-3 and UM-UC-5 cells.

Time	PC-3 IC_50_ (µM)		UM-UC-5 IC_50_ (µM)	
	Rilpivirine	Ritonavir	Ritonavir	Saquinavir
24 h	113.2	78.49	63.42	24.66
48 h	54.03	27.83	46.51	17.21
72 h	53.77	18.94	35.03	13.40

## Data Availability

The original contributions presented in this study are included in the article. Further inquiries can be directed to the corresponding author.

## Abbreviations

The following abbreviations are used in this manuscript:

RIT	Ritonavir
SAQ	Saquinavir
RPV	Rilpivirine
EFV	Efavirenz
ETV	Etravirine
PC-3	Human prostate cancer cell line
UM-UC-5	Human bladder cancer cell line
MRC-5	Human fetal lung fibroblast cell line
PI	Protease inhibitor
NNRTI	Non-nucleoside reverse transcriptase inhibitor
ROS	Reactive oxygen species
MTT	Thiazolyl blue tetrazolium bromide assay
DCFDA	2′,7′-Dichlorofluorescein diacetate
IC_50_	Half maximal inhibitory concentration
EC_50_	Half maximal effective concentration
DMEM	Dulbecco’s Modified Eagle’s Medium
FBS	Fetal bovine serum
PBS	Phosphate-buffered saline
DMSO	Dimethyl sulfoxide
SEM	Standard error of the mean
ANOVA	Analysis of variance
H_2_O_2_	Hydrogen peroxide
ATCC	American Type Culture Collection
ER	Endoplasmic Reticulum
